# Identification of furfural resistant strains of *Saccharomyces cerevisiae* and *Saccharomyces paradoxus* from a collection of environmental and industrial isolates

**DOI:** 10.1186/s13068-015-0217-z

**Published:** 2015-02-26

**Authors:** Sarah J Field, Peter Ryden, David Wilson, Stephen A James, Ian N Roberts, David J Richardson, Keith W Waldron, Thomas A Clarke

**Affiliations:** School of Biological Sciences, University of East Anglia, Norwich, NR4 7JN UK; Biorefinery Centre, Institute of Food Research, Norwich Research Park, Norwich, NR4 7UA UK; National Collection of Yeast Cultures, Institute of Food Research, Norwich Research Park, Norwich, NR4 7UA UK

**Keywords:** *Saccharomyces cerevisiae*, *Saccharomyces paradoxus*, Furfural, Furan, Ethanol, Lignocellulose

## Abstract

**Background:**

Fermentation of bioethanol using lignocellulosic biomass as a raw material provides a sustainable alternative to current biofuel production methods by utilising waste food streams as raw material. Before lignocellulose can be fermented, it requires physical, chemical and enzymatic treatment in order to release monosaccharides, a process that causes the chemical transformation of glucose and xylose into the cyclic aldehydes furfural and hydroxyfurfural. These furan compounds are potent inhibitors of *Saccharomyces* fermentation, and consequently furfural tolerant strains of *Saccharomyces* are required for lignocellulosic fermentation.

**Results:**

This study investigated yeast tolerance to furfural and hydroxyfurfural using a collection of 71 environmental and industrial isolates of the baker’s yeast *Saccharomyces cerevisiae* and its closest relative *Saccharomyces paradoxus*. The *Saccharomyces* strains were initially screened for growth on media containing 100 mM glucose and 1.5 mg ml^−1^ furfural. Five strains were identified that showed a significant tolerance to growth in the presence of furfural, and these were then screened for growth and ethanol production in the presence of increasing amounts (0.1 to 4 mg ml^−1^) of furfural.

**Conclusions:**

Of the five furfural tolerant strains, *S. cerevisiae* National Collection of Yeast Cultures (NCYC) 3451 displayed the greatest furfural resistance and was able to grow in the presence of up to 3.0 mg ml^−1^ furfural. Furthermore, ethanol production in this strain did not appear to be inhibited by furfural, with the highest ethanol yield observed at 3.0 mg ml^−1^ furfural. Although furfural resistance was not found to be a trait specific to any one particular lineage or population, three of the strains were isolated from environments where they might be continually exposed to low levels of furfural through the ongoing natural degradation of lignocelluloses, and would therefore develop elevated levels of resistance to these furan compounds. Thus, these strains represent good candidates for future studies of genetic variation relevant to understanding and manipulating furfural resistance and in the development of tolerant ethanologenic yeast strains for use in bioethanol production from lignocellulose processing.

**Electronic supplementary material:**

The online version of this article (doi:10.1186/s13068-015-0217-z) contains supplementary material, which is available to authorized users.

## Background

Dwindling world oil reserves and the need to develop motor fuels with a smaller carbon footprint has led to the explosion of research into sustainable fuels in the last 10 years [1]. Bioethanol is a very attractive biofuel to the automotive industry since it is miscible with petroleum gasoline and can be used in low concentration blends (<10%) in vehicles with no modifications [2]. It can be used effectively at higher concentrations with some power train modifications. In Brazil, dedicated E100 vehicles have been on the roads since 1979 [2]. The use of lignocellulosic waste materials such as straw as a source of glucose for microbial fermentation into bioethanol is of much interest as it negates the food versus fuel issue [3], and it has been estimated that 419 billion litres of bioethanol could be produced each year from crop wastage [4]. To release the glucose contained within lignocellulose, materials need to be pretreated by techniques such as steam explosion, followed by enzymatic hydrolysis. However, the high temperatures and acid conditions generated in these processes can lead to the dehydration of glucose and xylose to furfural and hydroxymethylfurfural (HMF), respectively, which are inhibitory to yeast growth and alcohol fermentation [5,6]. Furan compounds affect the yeast cell in a number of ways, including causing increased production of radical oxygen species and damage to DNA, protein and membranous structures. They also increase yeast sensitivity to osmotic and salt stress as well as specifically inhibiting key enzymes involved in carbon metabolism [7,8]. These toxic effects lead to an increased lag phase of growth and reduced ethanol production at low furan concentrations and cell death at high concentrations [9].

In order to protect themselves, yeasts reduce both furfural and HMF to their furyl acid or alcohol derivatives through NAD(P)H-dependent reductive pathways that utilise a range of aldehyde dehydrogenases involved in glycolysis and ethanol fermentation [9]. Under aerobic respiration *Saccharomyces cerevisiae* converts furfural to furoic acid [10], while under anaerobic fermentation the primary product is furfuryl alcohol [11]. These detoxification processes lead to a shortage of NADH, suggesting that furfural reduction competes for NADH and result in a decrease in cell growth and ethanol production [9,12,13]. For a detailed review centering on improving the resistance of yeast to furan by directed evolution or genetic manipulation, see Liu [14]. The aim of the present study has been to investigate yeast tolerance to furfural using a collection of over 70 environmental and industrial isolates of the baker’s yeast *S. cerevisiae* and its closest relative *S. paradoxus*. These strains were used in the *Saccharomyces* Genome Resequencing Project (SGRP), a landmark study in yeast population genomics [15]. One aim of this study was to assess their potential for inclusion in a strain improvement program.

## Results and discussion

### Growth and ethanol production of *S. cerevisiae* NCYC 2826 on wheat straw hydrolysate

**Figure 1:**
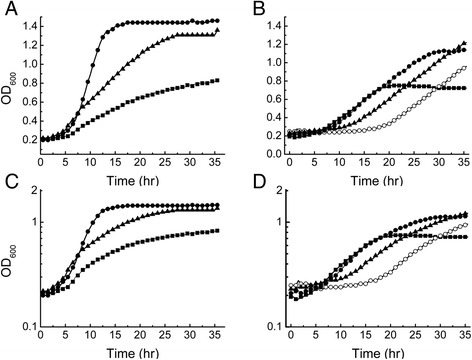
**Growth curves of** ***S. cerevisiae*** **NCYC 2826 measured by optical density at 600 nm.** Data shown are the average of three replicate experiments. **(A,** **C)** Growth using 10% wheat straw hydrolysate only (squares), 10% wheat straw hydrolysate and YNB (circles), or 10% wheat straw hydrolysate and 2.3 mg ml^−1^ urea (triangles). **(B,** **D)** Growth in media containing 2.3 mg ml^−1^ urea and initial wheat straw concentrations of 5% (squares), 10% (closed circles), 15% (triangle) or 20% (open circle). OD, optical density; hr, hour.

Figure 1A shows the growth of *S. cerevisiae* National Collection of Yeast Cultures (NCYC) 2826 grown at 30°C for 36 h in a culture containing a hydrolysate with a glucose concentration of 123 mM prepared as described in the ‘Methods’ section. The *S. cerevisiae* strain was chosen due to its reported high ethanol tolerance and robustness in industrial fermentations. Figure 1A shows that when *S. cerevisiae* NCYC 2826 was grown on wheat straw hydrolysate alone there was a slow growth rate *μ* of 0.036 h^−1^ and a final optical density (OD) of 0.8. Addition of Yeast nutrient base (YNB) to the media caused an increase in *μ* to 0.135 h^−1^ and a final OD of 1.5, while addition of 2.3 mg ml^−1^ urea to the wheat straw hydrolysate gave a *μ* of 0.99 h^−1^ and a final OD of 1.3. Previous studies have shown that urea supplements can increase ethanol production in yeast fermentation and that urea itself is an essential component in the most minimal yeast growth media [16,17]. Our results support these earlier findings, confirming the requirement of urea for near-optimal growth of yeast. After 36 h, the ethanol concentration in the cultures was measured and the yield of ethanol obtained from 123 mM glucose was approximately 90% of the total theoretical yield for all cultures. While ethanol was produced to a comparable yield under these three culture conditions, the growth was slower and the final optical density less on wheat straw hydrolysate than when either urea or YNB was added to the culture. This suggests that although glucose was available for fermentation, the hydrolysate did not contain sufficient nutritional elements to allow the culture to divide at its maximal rate and achieve optimal density.

**Figure 2:**
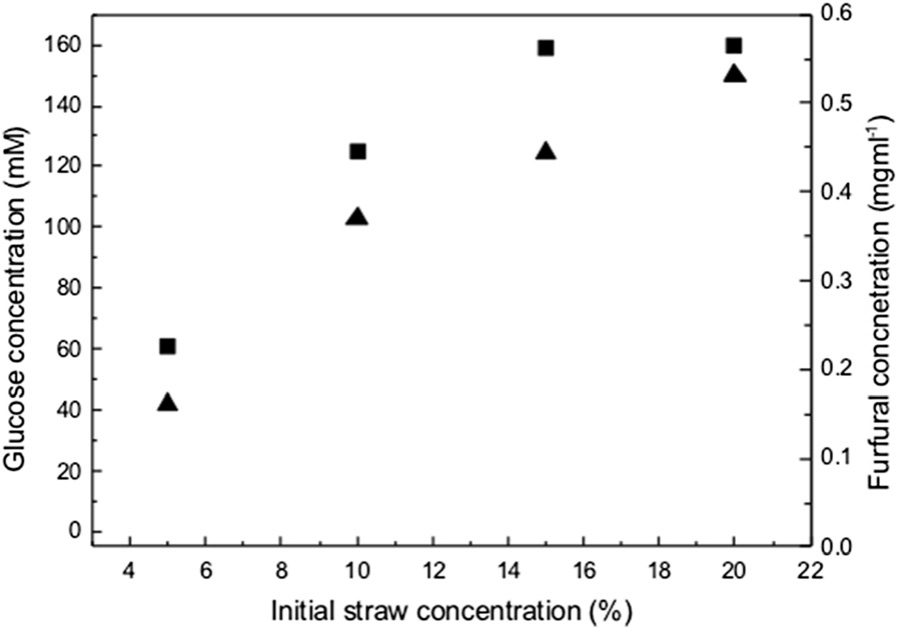
**Concentration of glucose (squares) and furfural (triangles) present in wheat straw hydrolysates made as described in the ‘** **Methods** **’ section with an increasing concentration of initial straw.**

To investigate the cause of the decreased cell growth on wheat straw hydrolysate, *S. cerevisiae* NCYC 2826 was grown on hydrolysate made using 5%, 10%, 15% and 20% starting straw concentration and supplemented with 2.3 mg ml^−1^ urea. Figure 1B shows that as the initial straw concentration increased, the lag phase of growth also increased to 20 h at an initial straw concentration of 20%. The final OD also increased as straw concentration increased, due to the increased concentrations of released glucose. The increased lag phase is characteristic of inhibition of growth by furan compounds often present in straw hydrolysates [14]. Analysis of the furan content of the hydrolysate showed that HMF content was negligible (data not shown) but the concentration of furfural present increased with initial straw concentration reaching 0.5 mg ml^−1^ at 20% initial straw content (Figure 2). These data suggest that growth of *S. cerevisiae* NCYC 2826 on wheat straw hydrolysate is limited by the concentration of furfural present in the hydrolysate.

### Analysis of SGRP strain set growth on furfural

In order to identify yeast strains that may be resistant to contaminating furfural, the SGRP strain set described in methods was grown in YNB, 100 mM glucose and the presence of 1.5 mg ml^−1^ furfural. Table 1 shows the analysis of tolerance of the SGRP strain set to 1.5 mg ml^−1^ furfural using the scoring system described in the ‘Methods’ section. A scoring system was required in place of average lag times as strain replicates that failed to grow did not have a measurable lag phase but still needed to be included in the dataset.

**Table 1:** **Furfural tolerance screen of SGRP yeast strains**

**Yeast strain**	**Score**	**Yeast strain**	**Score**
*S. cerevisiae* NCYC 3262	2.5 ± 1.5	*S. paradoxus* NCYC 3285	1.8 ± 1.7
*S. cerevisiae* NCYC 3314	2.5 ± 1.4	*S. paradoxus* NCYC 3336	2.5 ± 1.6
***S. cerevisiae NCYC 3451***	3.2 ± 1.2	*S. paradoxus* NCYC 3478	2.7 ± 1.5
*S. cerevisiae* NCYC 3460	2.8 ± 1.4	***S. cerevisiae NCYC 3290***	3.7 ± 0.5
*S. cerevisiae* NCYC 3471	2.8 ± 1.7	*S. cerevisiae* NCYC 3445	2.8 ± 1.5
*S. paradoxus* NCYC 3272	2.7 ± 1.5	*S. cerevisiae* NCYC 3455	2.3 ± 1.9
*S. paradoxus* NCYC 3281	2.8 ± 1.5	*S. cerevisiae* NCYC 3467	1.7 ± 1.9
*S. paradoxus* NCYC 3376	1.3 ± 1.2	***S. paradoxus NCYC 3277***	3.0 ± 1.3
*S. paradoxus* NCYC 3475	2.3 ± 1.6	*S. paradoxus* NCYC 3286	2.7 ± 1.5
*S. paradoxus* NCYC 3483	1.0 ± 1.3	*S. paradoxus* NCYC 3337	2.7 ± 1.5
*S. cerevisiae* NCYC 3265	2.0 ± 1.1	*S. paradoxus* NCYC 3479	2.7 ± 1.5
*S. cerevisiae* NCYC 3315	3.0 ± 1.6	*S. cerevisiae* NCYC 3311	2.5 ± 1.6
*S. cerevisiae* NCYC 3452	2.2 ± 1.3	*S. cerevisiae* NCYC 3447	2.8 ± 1.5
*S. cerevisiae* NCYC 3461	2.7 ± 1.5	*S. cerevisiae* NCYC 3456	2.8 ± 1.5
*S. cerevisiae* NCYC 3472	2.5 ± 1.5	*S. cerevisiae* NCYC 3468	2.3 ± 1.5
*S. paradoxus* NCYC 3274	1.0 ± 1.3	*S. paradoxus* NCYC 3278	2.7 ± 1.5
*S. paradoxus* NCYC 3282	1.2 ± 1.2	*S. paradoxus* NCYC 3287	2.2 ± 1.5
*S. paradoxus* NCYC 3317	2.0 ± 1.4	*S. paradoxus* NCYC 3377	1.8 ± 1.3
*S. paradoxus* NCYC 3476	2.0 ± 1.4	*S. paradoxus* NCYC 3480	1.3 ± 1.4
*S. paradoxus* NCYC 3484	2.2 ± 1.5	***S. cerevisiae NCYC 3312***	3.2 ± 1.2
*S. cerevisiae* NCYC 3266	2.5 ± 1.8	*S. cerevisiae* NCYC 3448	2.8 ± 1.5
*S. cerevisiae* NCYC 3318	2.3 ± 1.6	*S. cerevisiae* NCYC 3457	2.7 ± 1.4
*S. cerevisiae* NCYC 3453	2.5 ± 1.5	*S. cerevisiae* NCYC 3469	3.0 ± 1.6
*S. cerevisiae* NCYC 3462	2.5 ± 1.5	*S. paradoxus* NCYC 3279	2.3 ± 1.6
*S. cerevisiae* NCYC 3486	2.5 ± 1.8	*S. paradoxus* NCYC 3288	1.3 ± 1.5
*S. paradoxus* NCYC 3275	2.7 ± 1.5	*S. paradoxus* NCYC 3473	3.0 ± 1.6
*S. paradoxus* NCYC 3283	2.3 ± 1.6	*S. paradoxus* NCYC 3481	2.3 ± 1.6
*S. paradoxus* NCYC 3335	2.3 ± 1.5	*S. cerevisiae* NCYC 3313	2.3 ± 1.2
*S. paradoxus* NCYC 3477	1.5 ± 1.4	*S. cerevisiae* NCYC 3449	2.0 ± 1.3
*S. paradoxus* NCYC 3485	0.3 ± 0.8	*S. cerevisiae* NCYC 3458	2.8 ± 1.5
***S. cerevisiae NCYC 3284***	3.0 ± 1.3	*S. cerevisiae* NCYC 3470	2.8 ± 1.5
*S. cerevisiae* NCYC 3319	2.5 ± 1.4	*S. paradoxus* NCYC 3280	2.7 ± 1.5
*S. cerevisiae* NCYC 3454	2.5 ± 1.4	*S. paradoxus* NCYC 3289	1.0 ± 0.9
*S. cerevisiae* NCYC 3466	2.5 ± 1.5	*S. paradoxus* NCYC 3474	2.8 ± 1.5
*S. cerevisiae* NCYC 3487	2.3 ± 1.5	*S. paradoxus* NCYC 3482	1.7 ± 1.4
*S. paradoxus* NCYC 3276	2.5 ± 1.5		

Tolerance scores for *Saccharomyces* strains grown in plates containing YNB, 100 mM glucose and 1.5 mg ml^−1^ furfural. Strains were scored according to the duration of lag phase. A growth lag of 0 to 10 h was scored 4, 10 to 15 h scored 3, 15 to 20 h scored 2 and a lag phase of over 20 h scored 1 point. No observed growth was scored 0. Scores shown are the average ± standard deviation of six separate growth incubations. Strains with an average score greater than 2.9 and a standard deviation score less than 1.5 are in bold texts. NCYC, National Collection of Yeast Cultures; SGRP, *Saccharomyces* Genome Resequencing Project.

We had previously observed that increasing the inoculum into furfural containing cultures led to a decrease in the lag phase, presumably by maximising the quantity of viable yeast cells introduced to the medium leading to faster establishment of exponential phase of growth (data not shown). Therefore, for these experiments, a 5% inoculum volume of overnight culture was used. The data in Table 1 shows that the growth on replica plates was extremely variable and also strain dependent, demonstrating that a concentration of 1.5 mg ml^−1^ furfural is sufficient to distinguish furfural tolerance in strains of *S. cerevisiae* and *S. paradoxus*. When growth of strains was tested in YNB containing 100 mM glucose and either 2.0 or 3.0 mg ml^−1^ furfural, there was very little growth observed under either of these conditions from any of the strains analysed. Thus, it was decided to select strains using the 1.5 mg ml^−1^ data and to subject them to a more detailed furfural screen. Analysis of the data presented in Table 1 shows that overall *S. cerevisiae* strains grew better than *S. paradoxus* strains on 1.5 mg ml − 1 furfural. Nearly 20% of the *S. paradoxus* strains tested failed to receive a top mark in the scoring system while for *S. cerevisiae* this was less than 10%, and also reflected in the higher average overall score for *S. cerevisiae* of 2.5 ± 1.4 compared with 2.1 ± 1.4 for *S. paradoxus.* Within each strain group however, there was significant variation, with scores ranging from 1.7 to 3.7 for *S. cerevisiae* and from 0.3 to 3.0 for *S. paradoxus.* Strains that scored above 2.9 with a standard deviation of less than 1.5 were considered to show significant furfural tolerance. Consequently, *S. cerevisiae* strains NCYC 3284 (ex soil, USA), NCYC 3290 (ex bili wine, West Africa), NCYC 3312 (ex soil, The Netherlands) and NCYC 3451 (ex wort, Ireland), along with *S. paradoxus* NCYC 3277 (ex oak bark, UK) were examined further in a more detailed furfural screen.

### Effects of increasing concentrations of furfural on growth and ethanol production

**Figure 3:**
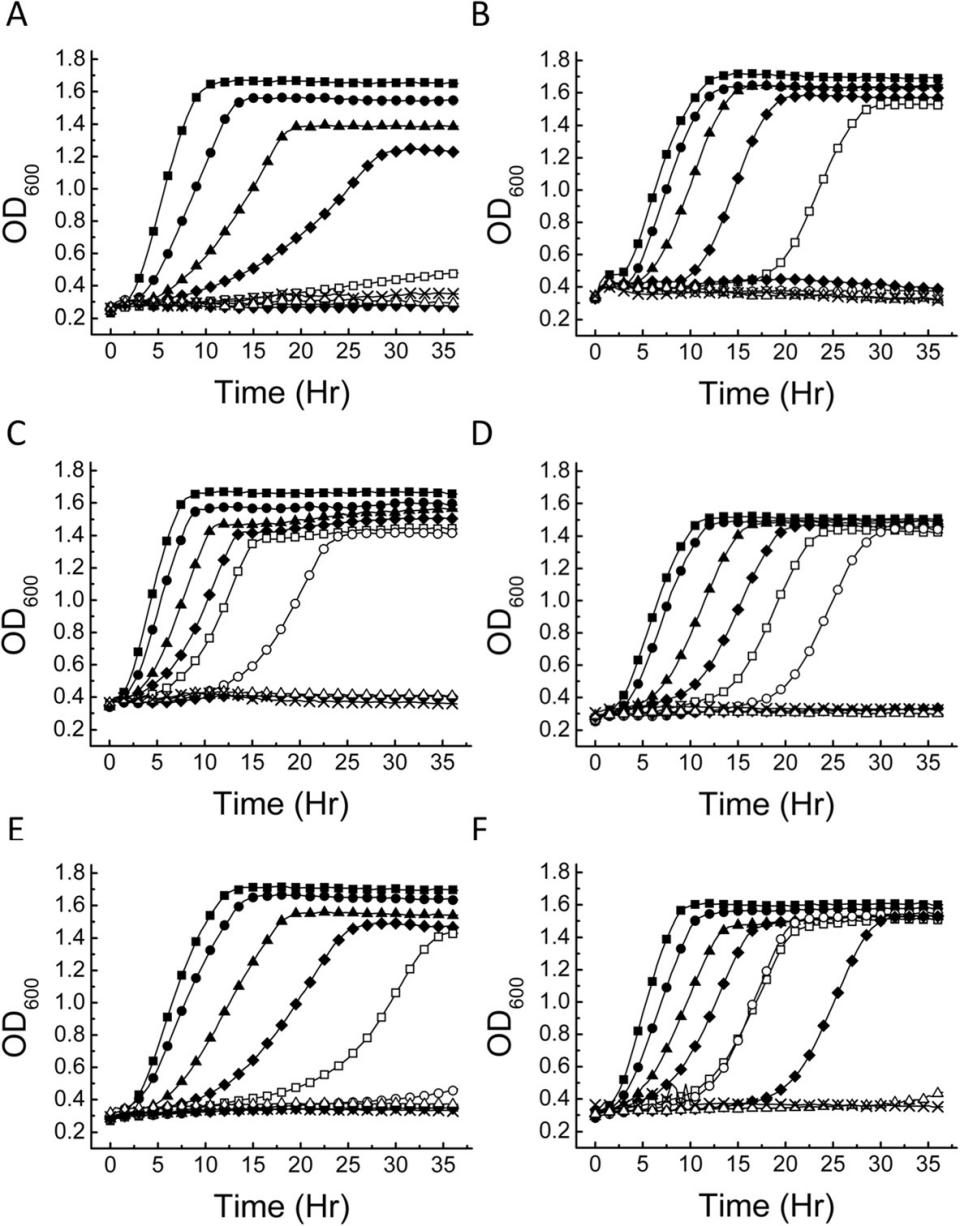
**Growth curves of** ***Saccharomyces*** **strains grown in yeast nutrient broth containing 100 mM glucose and furfural.** Data shown are the average of three replicate experiments. **(A)** *S. cerevisiae* NCYC 2826, **(B)** *S. paradoxus* NCYC 3277, **(C)** *S. cerevisiae* NCYC 3312, **(D)** *S. cerevisiae* NCYC 3290, **(E)** *S. cerevisiae* NCYC 3284 and **(F)** *S. cerevisiae* NCYC 3451. Media was supplemented with furfural at concentrations of 0.1 (squares), 0.5 (circles), 1.0 (triangles), 1.5 (diamonds), 2.0 (open squares), 2.5 (open circles), 3.0 (diamonds), 3.5 (open triangles) and 4.0 mg ml^−1^ furfural (crosses). OD, optical density; hr, hour.

Figure 3 shows growth in the presence of varying amounts of furfural (0.1 to 4.0 mg ml^−1^) for *S. cerevisiae* strains NCYC 3284, NCYC 3290, NCYC 3312 and NCYC 3451 and *S. paradoxus* strain NCYC 3277 identified in Table 1 from the SGRP strain set as having increased resistance to furfural. Additional file 1: Figure S1 shows the corresponding growth data plotted on a log scale. The control strain *S. cerevisiae* NCYC 2826 was also included for comparative purposes. For all six strains, as furfural concentration increased the growth curves begin to show increases in the lag phase as previously seen in growths containing furfural. All strains tested were able to grow on YNB supplemented with 100 mM glucose and 0.1 to 1.5 mg ml^−1^ furfural. *S. cerevisiae* NCYC 2826, our control strain, was only able to grow on up to 1.5 mg ml^−1^, which led to a 30% reduction in final OD when compared to growth on 0.1 mg ml^−1^ furfural. Table 2 shows that the ethanol production by NCYC 2826 under these conditions was considerably reduced compared to the approximately 90% yield observed when grown on YNB and glucose alone or on wheat straw hydrolysate. *S. cerevisiae* NCYC 2826 was isolated from grape must and so is unlikely to have evolved the ability to grow and ferment during exposure to furfural.

**Table 2:** **Ethanol yields from furfural tolerant** ***Saccharomyces*** **strains**

**Furfural concentration (mg ml** ^**−1**^ **)**	**NCYC 3451% ethanol yield**	**NCYC 3284% ethanol yield**	**NCYC 3290% ethanol yield**	**NCYC 3312% ethanol yield**	**NCYC 3277% ethanol yield**	**NCYC 2826% ethanol yield**
0.1	64 ± 5	86 ± 13	26 ± 8	41 ± 8	77 ± 19	59 ± 6
0.5	78 ± 8	89 ± 14	28 ± 5	75 ± 5	68 ± 7	38 ± 7
1.0	74 ± 7	82 ± 15	17 ± 3	54 ± 26	45 ± 10	62 ± 15
1.5	75 ± 14	37 ± 18	21 ± 1	35 ± 12	64 ± 10	47 ± 10
2.0	81 ± 15	61 ± 20	31 ± 2	62 ± 24	61 ± 17	-
2.5	78 ± 10	60 ± 19	30 ± 3	-	-	-
3.0	95 ± 15	-	-	-	-	-
3.5	-	-	-	-	-	-

*S. cerevisiae* strains NCYC 2826, 3451, 3284, 3290, 3312 and *S. paradoxus* strain 3277 were grown in triplicate on YNB, 100 mM glucose in the presence of the shown amount of furfural for 35 h, and ethanol yields were measured on each culture after 48 h. Ethanol was not measured on cultures that showed no signs of growth (indicated by a dashed line). NCYC, National Collection of Yeast Cultures.

In their population genomics study, Liti *et al*. [15] identified five well-defined, geographically isolated *S. cerevisiae* lineages (Malaysian, North American, Saké, West African and ‘Wine/European’) as well as many different recombinant (mosaic) strains of these lineages. From the results of the present study, it is apparent that furfural resistance is not a phenotypic characteristic specific to any one particular *S. cerevisiae* lineage. Of the four furfural resistant SGRP *S. cerevisiae* strains identified, NCYC 3284 (YPS128) belongs to the North American lineage, NCYC 3290 (DBVPG 6044) to the West African lineage, NCYC 3312 (DBVPG 1373) to the ‘Wine/European’ lineage, while NCYC 3451 (a single spore derivative of NCYC 361) is a recombinant strain.

*S. cerevisiae* NCYC 3451 displayed the greatest furfural resistance (Figure 3F, Additional file 1: Figure S1F) and was able to grow in the presence of up to 3.0 mg ml^−1^ furfural. Furthermore, ethanol production in this strain did not appear to be inhibited by furfural, with the highest ethanol yield (95 ± 15%; Table 2) achieved at a (furfural) concentration of 3.0 mg ml^−1^. As already mentioned, NCYC 3451 is a recombinant strain and has been shown to have a mosaic-like genome derived from at least three different lineages, namely Saké, West African and ‘Wine/European’ (Liti *et al*. [15]). Although recorded as being isolated from wort as a beer spoilage yeast, the highly complex genome structure of this strain would strongly suggest, although not proven, that it is of industrial origin (for example, a baking or brewing strain). Amongst the four remaining SGRP strains tested, *S. cerevisiae* strains NCYC 3290 and NCYC 3312 were both able to grow on 2.5 mg ml^−1^ furfural (Figure 3D,C, Additional file 1: Figure S1D and S1C, respectively), while *S. cerevisiae* NCYC 3284 (Figure 3E, Additional file 1: Figure S1E) and *S. paradoxus* NCYC 3277 (Figure 3B, Additional file 1: Figure S1B) could only grow on 2.0 mg ml^−1^ furfural. Overall, ethanol production in the five SGRP strains was not significantly affected by the presence of furfural. In fact, for NCYC 3312, the presence of 0.5 mg ml^−1^ furfural led to a notable increase in ethanol yield, from 41 ± 8% expected yield to 75 ± 5% (Table 2). This was also observed for the beer spoilage strain NCYC 3451, but to a lesser extent (only a 14% increase in yield; Table 2). Indeed, it has recently been shown that small amounts of furfuryl alcohol, a product of furfural dehydration in yeast, can actually lead to an increase in ethanol production [18].

## Conclusions

Production of bioethanol using lignocellulosic biomass is limited due to the presence of inhibitory furan compounds, and consequently furfural tolerant strains of *Saccharomyces* are required for lignocellulosic fermentation. Screening the 71 strains of the SGRP strain set for tolerance to 1.5 mg/mL furfural identified four strains of *S. cerevisiae* and one strain of *S. paradoxus* that appeared to have increased tolerance to furfural. These strains were revealed to be tolerant in concentrations of furfural up to 3.0 mg/mL, a concentration range often found in lignocellulosic extracts [19].

Although furfural resistance was not found to be a trait specific to any one particular lineage or population, three of the strains were from similar ecological sources. *S. cerevisiae* strains NCYC 3284 and NCYC 3312 were both isolated from soil, while *S. paradoxu*s NCYC 3277 was isolated from oak bark. In such environments/habitats, it is likely these yeasts would be constantly exposed to furfurals, from the ongoing natural degradation of lignocelluloses (for example, by white rot fungus), and would therefore develop elevated levels of resistance to these furan compounds. Thus, these strains represent good candidates for future studies of genetic variation relevant to understanding and manipulating furfuryl resistance and in the development of tolerant ethanologenic yeast strains for use in bioethanol production.

## Methods

### Preparation of wheat straw hydrolysate

Steam treatment was performed using a Cambi™ Steam Explosion Pilot Plant (Cambi, Asker, Norway). Winter wheat straw was obtained from Dixon Brothers, Rickinghall IP22 1LY, UK. Straw (500 g) was steam treated at 18.1 bar and 210°C for 10 min and exploded into 4 L warm (50°C) water. The slurry was centrifuged through a 100-μm nylon bolting cloth to a moisture content of 75.3% and stored at −40°C. The steam exploded straw was hydrolysed at substrate concentrations of 5%, 10%, 15%, and 20% in 50 mM NaOAc pH 5 with Biocatalysts enzyme cocktails (5% by mass of biomass of PDN N11/7 and 2.5% of PDN N11/9) in a rotary incubator (200 rpm) at 50°C for 42 h. The hydrolysate was recovered by centrifugation and boiled for 10 min to inactivate enzymes. The glucose monosaccharide concentration was measured with GOPOD reagent (Megazyme, Wicklow, Ireland).

### Growth of *S. cerevisiae* NCYC 2826 on wheat straw hydrolysate

*S. cerevisiae* NCYC 2826, a grape must isolate, was obtained from the NCYC, Norwich and stored in 25% glycerol at −80°C for use as a control strain. Cultures were revived by the addition of 100 μl of glycerol stock into 10 ml YM (yeast extract 3.0 gl^−1^, malt extract 3.0 gl^−1^, peptone 5.0 gl^−1^, glucose 10 gl^−1^) and incubated overnight at 30°C. Ninety six-well microtitre plates containing either 200 μl of wheat straw hydrolysate alone or supplemented with either yeast nutrient broth (Formedium, Norfolk, UK) or 2.3 mg ml^−1^ urea (Sigma Aldrich, Gillingham, UK) were inoculated with a 1% volume of the overnight culture. Plates were incubated at 30°C and shaken for 5 min before each reading. This ensured that cells were evenly distributed throughout the culture before the optical density was measured. Growth was monitored at 600 nm over 36 h by a FLOUstar omega multiwell plate reader (BMG Labtech, Bucks, UK). At the end of the experiment, cell supernatants were removed and stored at −20°C until required for ethanol analysis.

### Analysis of SGRP strain set by multiwell plate reader

The SGRP strain set (SGRP set 1) (Table 1), a collection of *S. cerevisiae* and *S. paradoxus* strains from a diverse variety of different ecological niches and geographical locations [15], was supplied by the NCYC, Norwich. The 71 *Saccharomyces* strains comprising this set were provided as glycerol stocks in a 96-well microtitre plate format and were stored at −80°C until required. A 50-μl aliquot of each glycerol stock (strain) was inoculated into 1 ml YM broth, and these cultures were incubated overnight at 30°C with shaking. A set of 96-well microtitre plates containing 200 μl YNB supplemented with 100 mM glucose and 1.5 mg ml^−1^, 2.0 or 3.0 mg ml^−1^ furfural (Sigma Aldrich, Gillingham, UK) were subsequently inoculated with a 5% volume of each overnight culture and growth was monitored using a FLOUstar omega multiwell plate reader.

The duration of lag phase was used to compare different strains, and in order to include strain replicates that failed to grow during the 36-h incubation, the lag phase was measured using the following scoring system: a lag phase of 0 to 10 h was scored 4, 10 to 15 h scored 3, 15 to 20 h scored 2 and a lag phase of over 20 h scored 1 point. No observed growth was scored 0.

### Furfural tolerance screen

Strains which scored highly in the initial analysis and our control strain NCYC 2826 were subjected to a furfural screen. Individual strains were grown in 5 ml YM at 30°C with shaking overnight. Subsequently, a 5% inoculum was added to 200 μl YNB supplemented with 100 mM glucose and either 0.1, 0.5, 1.0, 1.5, 2.0, 2.5, 3.0 or 3.5 or 4.0 mg ml^−1^ furfural. Growth was monitored at 600 nm over 30 h by a FLOUstar omega multiwell plate reader (BMG Labtech, Bucks, UK).

### Analysis of ethanol production

Ethanol production by *S. cerevisiae* and *S. paradoxus* strains was analysed using a Focus GC-FID Fisher Scientific UK Ltd, Loughborough, United Kingdom). Samples of supernatant were diluted in 1 ml analytical water (Fisher Scientific UK Ltd, Loughborough, United Kingdom) and sealed in 20 ml GC vials (Fisher Scientific UK Ltd, Loughborough, United Kingdom). Samples were incubated for 15 min at 70°C, 100 μl of headspace was injected onto a BAC1 column (Restek, Bellefonte, PA, USA) by a TriPlus headspace autosampler (Fisher Scientific UK Ltd, Loughborough, United Kingdom). Calibration curves were generated using HPLC grade ethanol (Sigma Aldrich, Gillingham, UK). Yields of ethanol were expressed as a percentage of the total theoretical yield based on the amount of glucose available.

## Additional file

Additional file 1:
**Semi-log plots of furfural tolerant** ***Sacchromyces*** **strains grown in yeast nutrient broth containing 100 mM glucose and furfural.**

## Abbreviations

HMF: hydroxymethylfurfural
NCYC: National Collection of Yeast Cultures
SGRP: *Saccharomyces* Genome Sequencing Project
YNB: yeast nutrient base
