# Supplementary material for: Identification of furfural resistant strains of Saccharomyces cerevisiae and Saccharomyces paradoxus from a collection of environmental and industrial isolates
Source: Biotechnol Biofuels. 2015 Feb 26;8:33. doi: 10.1186/s13068-015-0217-z (PMC4389715; doi:10.1186/s13068-015-0217-z)
Supplement: Additional file 1: — Semi-log plots of furfural tolerant Sacchromyces strains grown in yeast nutrient broth containing 100 mM glucose and furfural. [file 13068_2015_217_MOESM1_ESM.pdf]

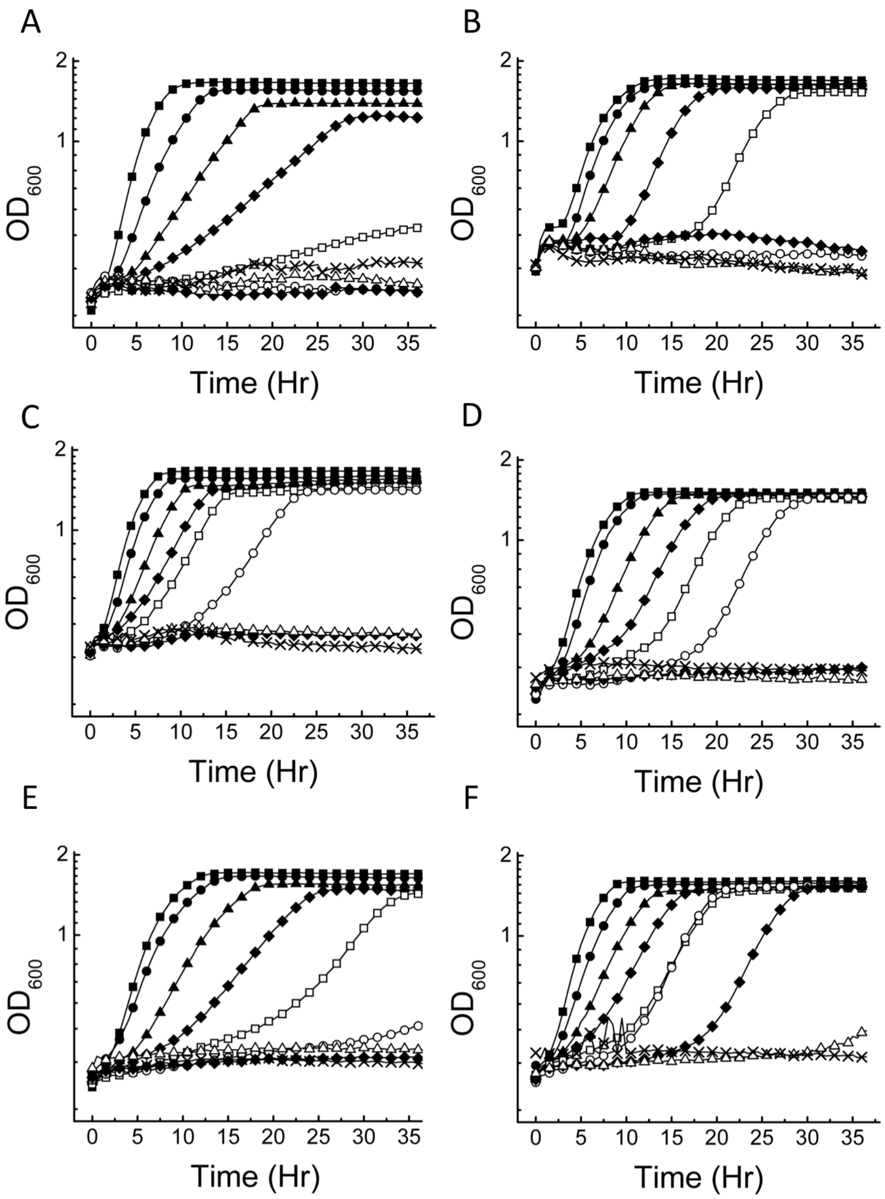

Figure 3 Growth curves of *Sacchromyces* strains grown in yeast nutrient broth containing 100 mM glucose and furfural. Data shown are the average of three replicate experiments and are plotted on a logarithmic scale. (**A**) *S. cerevisiae* NCYC 2826, (**B**) *S. paradoxus* NCYC 3277, (**C**) *S. cerevisiae* NCYC 3312, (**D**) *S. cerevisiae* NCYC 3290, (**E**) *S. cerevisiae* NCYC 3284 and (**F**) *S. cerevisiae* NCYC 3451. Media was supplemented with furfural at concentrations of 0.1 mg ml^{-1} (squares), 0.5 mg ml^{-1}(circles), 1.0 mg ml^{-1} (triangles), 1.5 mg ml^{-1} (diamonds), 2.0 mg ml^{-1} (open squares), 2.5 mg ml^{-1} (open circles), 3.0 mg ml^{-1} (diamonds), 3.5 mg ml^{-1} (open triangles) and 4.0 mg ml^{-1} furfural (crosses)
